# Morin hydrate promotes inner ear neural stem cell survival and differentiation and protects cochlea against neuronal hearing loss

**DOI:** 10.1111/jcmm.13005

**Published:** 2016-10-06

**Authors:** Qiang He, Zhanwei Jia, Ying Zhang, Xiumin Ren

**Affiliations:** ^1^ E.N.T DepartmentThe Second Hospital of Hebei Medical UniversityShijiazhuangChina

**Keywords:** morin hydrate, inner ear, neural stem cell, cochlea, hearing loss

## Abstract

We aimed to investigate the effect of morin hydrate on neural stem cells (NSCs) isolated from mouse inner ear and its potential in protecting neuronal hearing loss. 3‐(4,5‐dimethyl‐2‐thiazolyl)‐2,5‐diphenyl‐2‐H‐tetrazolium bromide (MTT) and bromodeoxyuridine incorporation assays were employed to assess the effect of morin hydrate on the viability and proliferation of *in vitro* NSC culture. The NSCs were then differentiated into neurons, in which neurosphere formation and differentiation were evaluated, followed by neurite outgrowth and neural excitability measurements in the subsequent *in vitro* neuronal network. Mechanotransduction of cochlea *ex vivo* culture and auditory brainstem responses threshold and distortion product optoacoustic emissions amplitude in mouse ototoxicity model were also measured following gentamicin treatment to investigate the protective role of morin hydrate against neuronal hearing loss. Morin hydrate improved viability and proliferation, neurosphere formation and neuronal differentiation of inner ear NSCs, and promoted *in vitro* neuronal network functions. In both *ex vivo* and *in vivo* ototoxicity models, morin hydrate prevented gentamicin‐induced neuronal hearing loss. Morin hydrate exhibited potent properties in promoting growth and differentiation of inner ear NSCs into functional neurons and protecting from gentamicin ototoxicity. Our study supports its clinical potential in treating neuronal hearing loss.

## Introduction

Neural stem cells (NSCs) are a type of multipotent cells in the neural system, which are able to self‐renew, proliferate and subsequently differentiate into neurons and glial cells at appropriate conditions 1. Neural stem cells possess potent clinical value particularly in treating neural damage and neurodegenerative diseases, where they were reported to become activated following neuronal injury, migrate towards sites of injury and replace the damaged neurons 2, 3. However, the underlying mechanisms governing the self‐renewal and differentiation of NSCs are still poorly understood. *In vitro* cultured primary NSCs may provide insights into the study of nervous system developments, where NSCs have been shown to be isolated and expanded *in vitro* in replacement stem cell therapies 1, 4. In this context, embryonic stem cells and pluripotent stem cells can be used to derive into early NSCs, which was reported to be able to proliferate following treatments with mitogens, and form neurospheres, which can be further differentiated into neural network *in vitro* 4.

Cochlear hair cells are the neuronal sensory receptors of the mammalian inner ear auditory system, which are innervated from neurons of the auditory nerves. Hair cells in the inner ear are terminally differentiated cells, hence are not able to self‐renew in cases of damage. Therefore, damages to the cochlear hair cells could cause compromised hearing sensitivity, even severe or complete hearing loss in some cases 5, which is a prevalent worldwide health problem. In this context, studies are urgently needed to further our understanding on the growth and differentiation of NSCs, in order to facilitate clinical treatments involving inner ear stem cells against neuronal hearing loss. Of note, a great number cases of neuronal hearing loss are caused by aminoglycosides, which is a type of Gram‐negative antibiotics that function to inhibit protein synthesis 6. Gentamicin is one type of such aminoglycosides and was reported to cause severe ototoxicity by damaging sensory hair cells 7.

Morin hydrate (3,5,7,2′,4′ pentahydroxyflavone) is a polyphenol compound found in *Castanea sativa* (sweet chestnut), *Prunus dulcis* (almond), *Morus alba L* (white mulberry), and other fruits 8, 9, and it is widely used in as dietary supplement and in traditional Chinese herbal medicine. Being yellow crystalline when extracted and purified, morin hydrate has exhibited a wide range of beneficial pharmacological effects, including anti‐cancer 10 and anti‐inflammatory activities 11, xanthine oxidase inhibitor property 12, preventing oxidation of low‐density lipoprotein 13, free radical scavenging activity 14 which in turn prevented DNA damage 15. However, to date, its function in NSC growth and differentiation, as well as in preventing neuronal hearing loss, has yet to be studied.

In this study, we first isolated NSCs from mouse inner ear and investigated the effect of morin hydrate on the *in vitro* NSC culture. We found that morin hydrate improved viability and proliferation, neurosphere formation and neuronal differentiation of inner ear NSCs and promoted *in vitro* neuronal network functions. In addition, using both *ex vivo* and *in vivo* ototoxicity models, morin hydrate exhibited protective roles against gentamicin‐induced neuronal hearing loss.

## Materials and methods

### Animal ethics

The protocol in this study was approved by the Committee on the Ethics of Animal Experiments and IACUC members of The Second Hospital of Hebei Medical University. All procedures involving animals in this study were carried out in strict accordance with the recommendations in the Guide for the Care and Use of Laboratory Animals of the National Institutes of Health. Sodium pentobarbital anaesthesia (40 mg/kg, i.p. injections) was used with all efforts made to minimize suffering in all surgeries performed in this study.

### Isolation and *in vitro* culture of mouse inner ear NSCs

Twelve early postnatal (P1–3) Balb/c mice were decapitated, followed by dissecting the temporal bone. The temporal bone was removed from the brain and transferred into ice‐cold Hank's balanced salt solution (Invitrogen, Pleasanton, CA, USA). After separating the otic capsule from the otic bulla, membranous labyrinth of cochlea is visible. The cochlear duct (organ of Corti, spiral ligament and stria vascularis) was carefully separated from the spiral ganglion concentrated modiolus. Previously established neurosphere assay protocol was used to isolate sphere‐forming stem cells 16. Briefly, 0.125% trypsin/ethylenediaminetetraacetic acid (EDTA) in PBS was used to treat spiral ganglia for 5 min. at 37°C. A cocktail of 10 mg/ml soybean trypsin inhibitor (Worthington, Lakewood, NJ, USA) and 1 mg/ml DNase I (Worthington) in DMEM and Nutrient Mixture F‐12 (DMEM/F12; Sigma‐Aldrich, St. Louis, MO, USA) was used to neutralize the above trypsin solution. Homogenous single‐cell suspension was obtained by firstly mechanically dissociating with pipette tip and passing through 70‐μm cell strainer (BD Falcon, Franklin Lakes, NJ, USA). The cells were cultured in 2 ml of DMEM/F12 media supplemented with B27 (Invitrogen), N2 (Invitrogen), 50 ng/ml heparan sulphate, 50 ng/ml insulin‐like growth factor‐1 20 ng/ml, epidermal growth factor (EGF), 10 ng/ml basic fibroblast growth factor (FGF) (all growth factors are from Sigma‐Aldrich) and 50 μg/ml ampicillin in poly‐HEMA‐coated suspension culture six‐well plates (Sigma‐Aldrich). Sphere size was recorded as the maximum sphere diameter. Cells between passages 3 and 5 were used in this study.

### Differentiation of inner ear NSCs

After 3–5 days of culture, neurospheres were harvested and single‐cell dissociated using Accumax (PAA Laboratories, Shanghai, China) and re‐suspended to a concentration of 2 × 10^4^ cells/ml. The cells were then differentiated in 100 μl of DMEM/F12 per well supplemented with N2, B27, 50 ng/ml NT‐3 (both from R&D Systems Minneapolis, MN, USA), 50 ng/ml brain‐derived neurotrophic factor, and 50 μg/ml ampicillin in a 0.1% gelatin‐coated 4‐well tissue culture plates. Media change was done every other day in a way that three quarters of the medium was removed and replaced with equal volume of fresh and pre‐warmed medium.

### Cell viability assay

Viability was measured using the MTT assay. Briefly, 10^4^ cells were seeded in 96‐well plates at assay specific concentration followed by 24‐hr incubation, and 10 μl of 5 mg/ml MTT solution was added to each well followed by 4‐hr incubation at 37°C in the dark. After incubation, the media with MTT was removed and 100 μl DMSO was added to each well to dissolve formazan crystals. The reduction of MTT was evaluated by absorbance at 570 nm in a microplate reader.

### Cell proliferation assay

Neural stem cell proliferation was measured using the bromodeoxyuridine (BrdU) incorporation assay kits (Millipore, Billerica, MA, USA). Briefly, 10^4^ cells were seeded in each well of a 96‐well plate with morin with indicated concentrations followed by 48 hrs of incubation. Bromodexyuridine was added at hour 46 of incubation. The following steps of the assay were carried out according to the manufacturer's instructions.

### RT‐PCR

RNeasy MiniPrep Kit (Qiagen, Valencia, CA, USA) was used to extract total RNA from the samples. Superscript II First‐Strand Synthesis Kit (Life Technologies, Pleasanton, CA, USA) was used to reverse‐transcribed 1 μg of total RNA from each sample according to the manufacturer's instructions. GAPDH was used as internal control to normalize all data, and the results were presented as relative expression. The following primer pairs were used in the study: microtubule‐associated protein‐2 (*MAP2*) forward 5′‐CCA CCT GAG ATT AAG GAT CA‐3′ and reverse 5′‐GGC TTA CTT TGC TTC TCT GA‐3′, *GAPDH* forward 5′‐CGA CTT CAA CAG CAA CTC CCA‐3′ and reverse 5′‐GGG TGG TCC AGG GTT TCT TAC‐3′.

### Western blot

Sample cells were washed twice with ice‐chilled 1× PBS followed by lysis in Radio‐Immunoprecipitation Assay (RIPA) buffer which contains 1 mM Na_3_VO_4,_ 1 mM phenylmethanesulfonyl fluoride (PMSF), 150 mM NaCl, 1 mM EDTA, 1 mg/ml aprotinin, 1 mg/ml leupepetin, 1% Nonidet P‐40 and 50 mM Tris‐HCl (pH 7.4). Cell lysates were sent to electrophoresis in SDS‐PAGE gel and transferred to nitrocellulose membrane. Anti‐MAP2 or anti‐growth‐associated protein‐43 (GAP43) antibodies (Sigma‐Aldrich) were used to immunoblot the membrane for 2 hrs, followed by incubation of HRP‐conjugated secondary antibody (Santa Cruz, Dallas, TX, USA) for 1 hr. Electro‐chemi‐luminescence (ECL) detection reagents were used to detect specific bands on the membrane.

### Calcium signalling assays

Sample cells were washed twice with standard solutions containing contain 5 mM KCl, 10 mM N′‐a‐hydroxythylpiperazine‐N′‐ ethanesulfanic acid (HEPES), 150 mM NaCl, 1 mM MgCl_2_, 2 mM CaCl, 10 mM D‐glucose with a pH of 7.3, followed by adding pluronic F‐127 (Sigma‐Aldrich, St. Louis, MO, USA) and 2.5 μM fluo‐8‐AM (Dojindo Laboratories, Kumamoto, Japan) to incubation for 45 min. The fluo‐8‐AM solution was then removed and standard solution was added and incubated for another 30 min. Cells were visualized and imaged using an Olympus scanning confocal microscope. The frequency of calcium oscillations was recorded as number of spikes per minute. The relative fluorescence amplitudes of the calcium spikes were calculated by normalizing the fluorescence of each cell (∆F) to the average fluorescence intensity (F) as ∆F/F.

### Isolation and *ex vivo* culture of mouse cochlea

Early postnatal (P1–3) Balb/c mice were killed to obtain the cochlear duct as described above. Cochlear tissue isolation was performed according to previously published procedures 17, 18, which a flat cochlear surface preparation was obtained by removing most basal cochlear segment. The cochlear tissue was then cultured on SPI filter membranes (Spi Supplies) in DMEM‐F12 (Invitrogen) basal media supplemented with B27 (Invitrogen), 5 ng/ml EGF (Sigma‐Aldrich), 2.5 ng/ml FGF2 (NIH, Bethesda, MD, USA) and 100 U/ml Penicillin (Sigma‐Aldrich). All cell cultures were incubated 5% CO_2_/20% O_2_‐humidified incubator.

### Mechanotransduction measurement

Morin hydrate (M4008; Sigma‐Aldrich) was supplemented at the concentrations as indicated, the same media without morin was used as control. To ensure concentration of morin and other growth factors, all media were changed daily. Isolated cochlea were divided into three experimental groups (*n* = 10 each): (*i*) control, 0 mM gentamicin + 0 mg/l morin hydrate; (*ii*) GM, 3 mM gentamicin + 0 mg/l morin hydrate; and (*iii*) GM+morin, 3 mM gentamicin + 2 mg/l morin hydrate. After 24 hrs of respective treatments, they were then subjected to mechanotransduction measurements following previously established methods 19, 20. Briefly, utricles and cochleae were separated and mounted on glass coverslips and viewed using Axioskop FS upright microscope (Zeiss, Oberkochen, Germany) equipped with a 633 water‐immersion objective and differential interference contrast optics. Electrophysiological recordings were done in solutions containing 5.6 mM D‐glucose, 5.8 mM KCl, 0.9 mM MgCl_2_, 137 mM NaCl, 0.7 mM NaH_2_PO_4_, 10 mM HEPES and 1.3 mM CaCl_2_, with vitamins (1:100) and amino acids (1:50) as in MEM (Invitrogen, Pleasanton, CA, USA) and a pH of 7.4 (311 mOsm/kg). Electrodes (2–4 MΩ) recording was pulled from R‐6 glass (King Precision Glass, Claremont, CA, USA) and filled with solution containing: 0.1 mM CaCl_2_, 5 mM EGTA‐KOH, 2.5 mM MgCl_2_, 2.5 mM Na_2_ATP, 5 mM HEPES and 135 mM KCl, pH 7.4 (284 mOsm/kg). Axopatch 200B (Molecular Devices, Sunnyvale, CA, USA) was used for utricles, and Multiclamp 700A amplifier (Molecular Devices) was used for cochleae in patch clamp experiment. Cells were held at a physiologically relevant holding potential of 100 mV. The currents were filtered using a low‐pass Bessel filter at 2–5 kHz and digitized using a 12‐bit acquisition board (Digidata 1322A or 1440A) at above 20 kHz, and data were recorded with pClamp 8.2 software (Molecular Devices). A stiff glass probe mounted on a PICMA chip piezo actuator was used to deflected inner hair bundles. The actuator is driven by a 400 mA ENV400 amplifier (Piezosystem Jena, Jena, Deutschland) filtered with at 10–40 kHz with an 8‐pole Bessel filter in order to eliminate residual pipette resonance.

### *In vivo* ototoxicity mouse model

The *in vivo* ototoxicity model was established by supplying gentamicin and/or morin into the mouse inner ear according to established procedures 21. Mice were randomly divided equally into three experimental groups (*n* = 10 each): (*i*) control, 0 mg/kg gentamicin + 0 mg/kg morin hydrate; (*ii*) GM, 40 mg/kg gentamicin + 0 mg/kg morin hydrate; and (*iii*) GM+morin, 40 mg/kg gentamicin + 10 mg/kg morin hydrate.

### Auditory brainstem responses measurement

Auditory brainstem responses (ABR) threshold measurements were carried out according to previously published methods 22. Animals were anaesthetized using ketamine (40 mg/kg) and xylazine (10 mg/kg) mixture on a heating pad before ABR measurement. The needle electrodes for reference, ground and active were inserted beneath the post‐measured auricle, the sacrococcygeal region and the calvaria accordingly. After amplification and filtration, the responses to 1024 click presentations were synchronously averaged. Each stimulus was presented at 100 dB sound pressure level (SPL) initially. A decreasing stimulus intensity at 5‐dB per step is used till ABR waveform is not visually discernible. The lowest level of the stimulus that produced a visually detectable response was defined as ABR threshold.

### Distortion product optoacoustic emissions amplitude measurement

In a quiet room, optoacoustic emission (OAE) measurements were first repeatedly performed on the ears of mice with otomicroscopically inspected normal ear canals and tympanic membrane. Only those mice with normal and reproducible OAEs before the treatment with gentamicin and/or morin hydrate were accepted for this study. Then, the distortion product optoacoustic emissions (DPOAEs) were recorded from the mice in each experimental groups using the commercial OAE apparatus of ILO‐96 cochlear emission analyser (Otodynamics Ltd., London, UK). Following anaesthetic treatment, an earphone probe was inserted into the outer ear canal of the mice using a plastic adapter and sealed, through which the primary tones were introduced. Distortion product optoacoustic emissions were continuously recorded as SPL by stimuli with constant intensity of 65 dB at different frequencies (1000, 2000, 3000, 4000 and 5000 Hz), until the responses reached the highest level. Measurement was terminated when there was no further increase in DPOAE amplitude levels.

### Statistical analysis

Values were shown as mean ± S.D. All *in vitro* cell culture experiments were repeated at least three times independently, while *ex vivo* cochlea explant and *in vivo* animal ototoxicity experiments were performed in group size of 10. Student's *t*‐test or one‐way anova were employed for statistical comparisons, where appropriate. Significant anova effect was followed by Tukey's post hoc test, where *P* < 0.05 was considered as statistically significant.

## Results

### Morin hydrate enhanced viability and proliferation of mouse inner ear NSCs *in vitro* culture

**Figure 1:**
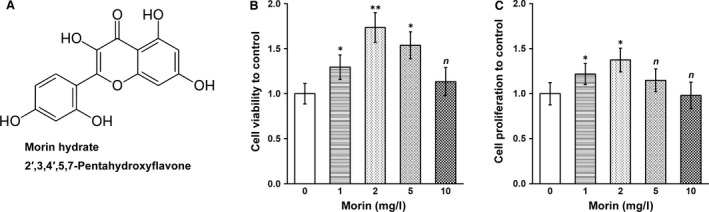
Morin was able to enhance viability and proliferation of inner ear NSCs. (**A**) Chemical structure of morin hydrate. (**B** and **C**) Inner ear NSCs were treated with increasing doses of morin as indicated, followed by MTT and BrdU incorporation assays to determine their (**B**) viability and (**C**) proliferation relative to control treatment (0 mg/l). Values were shown as mean ± S.D. *P < 0.05, **P < 0.01, *n* not significant, compared to 0 mg/l morin as control.

In order to investigate the effect of morin hydrate (Fig. 1A) on the inner ear NSCs, we added increasing concentrations of morin hydrate (1, 2, 5 and 10 mg/l) into the culture media, respectively, and performed MTT assay after 24 hrs to assess the viability. Compared with control treatment (0 mg/l morin hydrate), morin hydrate at 1 and 2 mg/l significantly enhanced the viability of the *in vitro* cultured inner ear NSCs, with 2 mg/l dose displaying higher enhancement (Fig. 1B). However, when morin concentration was further increased to 5 and 10 mg/l, the NSC viability started to decline, eventually back to the same level as control treatment. Results from BrdU assay, a cell proliferation measurement, displayed a very similar dose‐dependent trend after 24 hrs incubation with increasing doses of morin hydrate, in which 2 mg/l exerted the most beneficial effect on NSC proliferation (Fig. 1C). Therefore, 2 mg/l morin hydrate was chosen as the optimal *in vitro* dose for the rest of our experiments on the inner ear NSC culture.

### Morin hydrate improved neurosphere formation of inner ear NSCs

**Figure 2:**
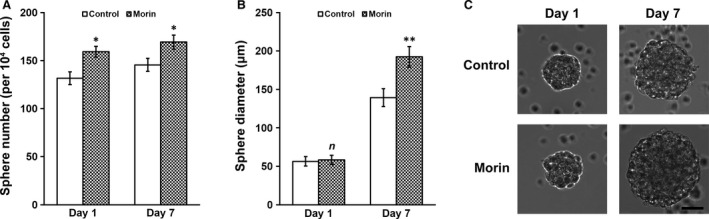
Morin was able to improve formation and growth of neurospheres of inner ear NSCs. Inner ear NSCs were cultured for 7 days in the absence or presence of 2 mg/l morin, followed by determination of (**A**) number and (**B**) diameter of the formed neurospheres at days 1 and 7. Values were shown as mean ± S.D. *P < 0.05, **P < 0.01, *n* not significant, compared with control. (**C**) Representative images of neurospheres at days 1 and 7, in the absence and presence of 2 mg/l morin; scale bar 50 μm.

We next extended the morin hydrate treatment (2 mg/l) to 7 days in the inner ear NSC culture in basal media containing growth factors and followed the formation and growth of neurospheres. As early as 24 hrs into the incubation, we observed significantly higher number of neurospheres in the morin hydrate‐treated culture than control culture, and this difference persisted throughout the 7‐day experiment (Fig. 2A). However, average sphere sizes were approximately the same between morin hydrate and control culture on day 1 (Fig. 2B). However, growth of morin hydrate‐treated spheres soon surpassed that of control, and by the end of day 7, diameter of morin hydrate‐treated neurospheres was markedly longer than the control (Fig. 2B and C). These above results indicated that 2 mg/l morin hydrate was able to improve neurosphere formation and growth of the inner ear NSC culture *in vitro*.

### Morin hydrate enhanced neuronal differentiation of inner ear NSCs

**Figure 3:**
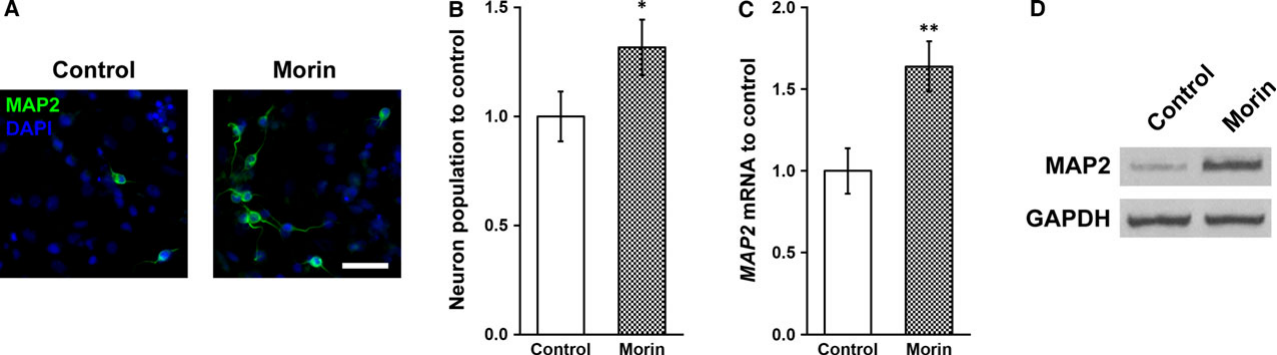
Morin was able to enhance the inner ear NSCs differentiation into neurons. Inner ear NSCs were cultured in differentiation media in the absence or presence of 2 mg/l morin for 2 weeks, followed by (**A**) immunofluorescence staining of MAP2 (green) and nuclear staining of DAPI (blue), as well as measurements of (**B**) neuron density, (**C**) mRNA and (**D**) protein levels of MAP2, scale bar 100 μm. Values were shown as mean ± S.D. *P < 0.05, **P < 0.01, compared with control.

We harvested neurospheres from the culture and subjected them to differentiation conditions, in the presence and absence of 2 mg/l, respectively, for 2 weeks. After differentiation, the derived neurons in the culture can be identified by the neuron‐specific protein marker MAP2 (Fig. 3A). We found significantly higher number of MAP2‐positive neurons in the morin hydrate‐treated culture than in control (Fig. 3B). In consistent with the increased immunofluorescent staining or MAP2 in morin hydrate‐treated culture (Fig. 3A), the mRNA and protein levels of MAP2 were also found to be greatly elevated by morin hydrate treatment (Fig. 3C and D), further confirming the enhanced neuronal differentiation capacity of the inner ear NSCs by morin hydrate.

### Morin hydrate promoted neural network formation *in vitro*

**Figure 4:**
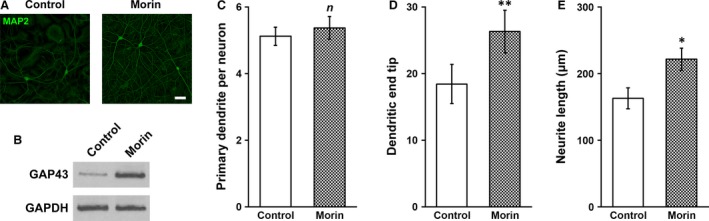
Morin was able to promote neurite outgrowth of inner ear NSC‐derived neurons. Inner ear NSCs were differentiated for 3 weeks in the absence or presence of 2 mg/l morin, followed by (**A**) immunofluorescence staining of MAP2 (green), (**B**) Western blot analysis of GAP43 protein expression levels, as well as measuring (**C**) primary dendrite number per cell, (**D**) number of dendritic end tip number, and (**E**) neurite length, scale bar 100 μm. Values were shown as mean ± S.D. *P < 0.05, **P < 0.01, *n* not significant, compared to control.

Next with MAP2 immunofluorescence, we could observe neurite formation *in vitro*, and simple visual inspection indicated that the neural network appeared to be more complex in the morin hydrate‐treated culture than control (Fig. 4A). To confirm this observation, we examined the level of GAP43 in the culture, which serves as the marker of neurite outgrowth 23, and found that GAP43 protein was apparently increased in the NSC‐derived neuron culture by morin hydrate treatment (Fig. 4B). Further validation of the improved complexity of the neural network following morin hydrate treatment came from the assessment of network morphological characteristics, including the number of primary dendrites, dendritic end tips and neurite length. The average number of primary dendrites spurned per neuron was not affected by morin hydrate treatment (Fig. 4C), suggesting limited effect of morin hydrate on individual neuron level. In contrast, the number of dendritic end tips and average length of the neurite were both significantly increased by morin hydrate (Fig. 4D and E), which clearly indicated a morphologically more complex neural network with higher and farther inter‐neuron connections.

### Morin hydrate increased excitability of the *in vitro* neural network

**Figure 5:**
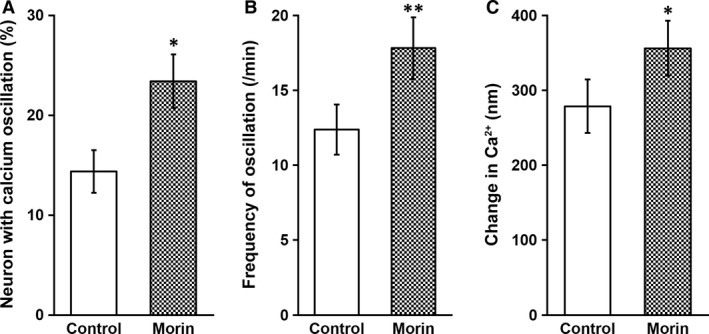
Morin was able to increase neural excitability of inner ear NSC‐derived neuronal network culture. Inner ear NSCs were differentiated for 3 weeks in the absence or presence of 2 mg/l morin, followed by measurements of (**A**) percentage of neurons with calcium oscillation, (**B**) frequency of oscillation and (**C**) change in calcium concentration. Values were shown as mean ± S.D. *P < 0.05, **P < 0.01, compared with control.

Calcium activity plays an essential role in mature neurons, where it regulates both neural excitability and synaptic plasticity. In order to assess the effects of morin hydrate on the functionality of the *in vitro* neural network, we conducted calcium signalling assay, in the absence and presence of morin hydrate. Calcium oscillations were visualized by fluo‐8‐AM staining, as spontaneous and rhythmic spikes of fluorescent signals 24. As expected, morin hydrate treatment greatly increased the number of neurons with calcium oscillation (Fig. 5A), as well as average oscillating frequency per neuron (Fig. 5B), both of which indicated higher neural excitability throughout the network. This elevated neural network excitability was further confirmed by significantly higher extent in overall concentration changes of calcium in the morin hydrate‐treated culture than that of the control network (Fig. 5C).

### Morin hydrate protected mechanotransduction potential of isolated mouse cochlea against ototoxicity

**Figure 6:**
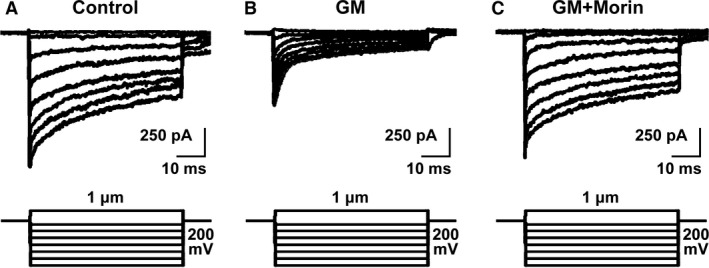
Morin was able to protect mechanotransduction potential of isolated mouse cochlea explant from gentamicin‐induced ototoxicity. Isolated cochlea hair cells were treated with (**A**) control (0 mM gentamicin + 0 mg/l morin hydrate), (**B**) GM (3 mM gentamicin + 0 mg/l morin hydrate) and (**C**) GM+Morin (3 mM gentamicin + 2 mg/l morin hydrate), respectively, followed by measurements of mechanotransduction currents.

With the above results, we have shown that morin hydrate was able to promote growth, differentiation and functionality of *in vitro* cultured inner ear NSCs and subsequently formed neural network, respectively. We next asked the question these beneficial effects of morin hydrate could be reflected in the protection against ototoxicity in more complex models. In this context, we used the isolated intact cochlea tissue from mouse inner ears and cultured them *ex vivo*, and treated them with gentamicin to induce ototoxicity, in the presence and absence of morin hydrate, followed by measuring their mechanotransduction potentials. First of all, baseline mechanotransduction response currents were recorded in control (0 mM gentamicin + 0 mg/l morin hydrate) treated cochlea explant (Fig. 6A). On the contrary, 3 mM gentamicin treatment severely abrogated mechanotransduction (Fig. 6B). However, when the cochlea *ex vivo* culture was treated with 3 mM gentamicin and 2 mg/l morin hydrate simultaneously, mechanotransduction response was restored to comparable levels as control (Fig. 6C). Our *ex vivo* model using isolated mouse cochlea tissue, therefore, supported the protective role of morin hydrate against gentamicin‐induced ototoxicity.

### Morin hydrate prevented gentamicin ototoxicity *in vivo*

**Figure 7:**
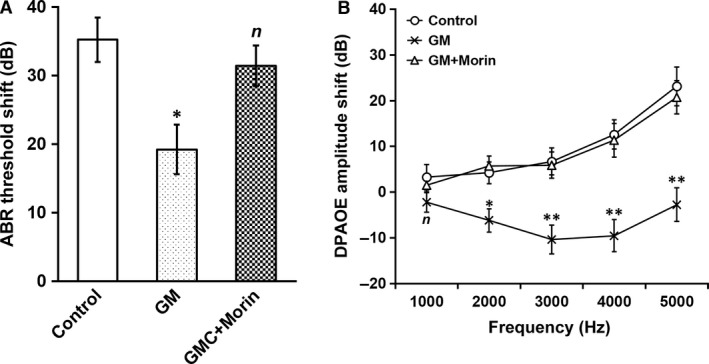
Morin was able to protect mice from gentamicin‐induced neuronal hearing loss *in vivo*. Mice were divided into three experimental groups (*n* = 10 each) and treated with control (0 mg/kg gentamicin + 0 mg/kg morin hydrate), GM (40 mg/kg gentamicin + 0 mg/kg morin hydrate) and GM+Morin (40 mg/kg gentamicin + 10 mg/kg morin hydrate), respectively, followed by measurements of (**A**) ABR threshold shift and (**B**) DPOAE amplitude shift. Values were shown as mean ± S.D. *P < 0.05, **P < 0.01, *n* not significant, compared with control.

We then evaluated the conductive hearing ability of mice, by measuring their ABRs threshold and DPOAE amplitude, both of which are quantitative indicators of conductive hearing loss resulted from sensorineural impairment *in vivo* 25. Similar as *ex vivo* ototoxicity model, we first established baseline ABR threshold and DPOAE amplitude shifts in mice from control treatment (0 mg/kg gentamicin + 0 mg/kg morin hydrate) group (Fig. 7A and B, control). Next, 40 mg/kg gentamicin was administered into the inner ear of the mice, followed by recordings of their ABR threshold and DPOAE amplitude. Gentamicin expectedly caused severe damage to the conductive hearing ability of the animals (Fig. 7A and B, GM). Importantly, when the animals were treated with 40 mg/kg gentamicin and 10 mg/kg morin hydrate at the same time, the adverse effect of gentamicin on their hearing was neglectible (Fig. 7A and B, GM+morin). Using this *in vivo* ototoxicity mouse model, we further demonstrated that morin hydrate possessed potent protective property against gentamicin‐induced conductive hearing loss.

## Discussion

In this study, we first discovered that morin hydrate, a natural polyphenol with many potent beneficial properties, is able to promote growth and proliferation of isolated mouse inner ear NSC culture, enhance the excitability in the subsequently formed *in vitro* neural network. We then established two gentamicin‐induced ototoxicity models to further elucidate the function of morin hydrate in preventing hearing loss. This first model was isolated intact mouse cochlea tissue explant, with which we investigated their mechanotransduction potential, following treatments with gentamicin and/or morin hydrate. The results indicated that gentamicin indeed severely attenuated the mechanotransductive ability in the *ex vivo* cultured cochlea tissues, and morin hydrate was able to alleviate such adverse effect of gentamicin and restored the mechanotransduction to levels comparable to control treatment. Next, we proceeded to treat mouse with gentamicin to establish an animal ototoxicity model, and further evaluated whether morin hydrate was able to exert similar protective roles *in vivo*. In both the measurements of ABR threshold and DPOAE amplitude shifts, morin hydrate displayed potent effects in restoring gentamicin‐induced hearing loss.

Morin hydrate has been reported to exhibit many other clinical benefits. For instance, in a rat chronic colitis model, morin hydrate was able to down‐regulate factors involved in the inflammatory response thereby exert anti‐inflammatory effect 11. It also possesses potent anti‐oxidant and free radical scavenging abilities 14, 15. Moreover, morin hydrate was found to attenuate CD36 expression, as well as oxidative modification of low‐density lipoproteins and their subsequent update, in U937‐derived macrophages 13. In addition, in an oxonate‐induced hyperuricemic rat model, morin hydrate was found to act as a hypouricemic agent and xanthine oxidase inhibitor 12. It is, therefore, interesting to uncover the underlying molecular mechanisms, in order to promote its precise and controlled clinical application.

In the context of our current study, similar as other aminoglycosides, gentamicin is found to accumulate in the mitochondria of hair cells and cause excessive production of reactive oxygen species 26, which is likely to be the cause of aminoglycoside‐induced hearing loss 27. Indeed, early reports suggested that gentamicin‐induced oxidative stress, and in turn apoptosis in hair cells of various species 28, 29, 30. Of particular interest to our current work, morin hydrate exhibited anti‐apoptotic functions in a recent study conducted in a rat gastropathy model, through modulation of the nuclear factor‐kappaB (NF‐κB) pathway 31. In contrast, Manna *et al*. 10 reported that morin hydrate could up‐regulate apoptosis by antagonizing NF‐κB‐regulated gene expressions. Although seemingly contradictory, the above reports highlighted the importance of understanding the molecular functions of morin hydrate, especially involving apoptosis. We speculated that the anti‐apoptotic property of morin hydrate could be exploited in the prevention against gentamicin‐induced ototoxicity. Investigations are currently underway to explore the possible mechanisms responsible for the observed beneficial role of morin hydrate in preventing gentamicin‐induced hearing loss, using our *ex vivo* and *in vivo* ototoxicity models.

In conclusion, we hereby presented the very first report on the role morin hydrate, both as a promoter of NSCs growth and differentiation and a protective agent against gentamicin‐induced ototoxicity. Morin hydrate is a naturally existed and abundant safe polyphenol and gives its observed functions in this study, and detailed investigations on its cellular and molecular roles, as well as its pharmacokinetics in animals, are warranted, to advance its potential clinical applications, particularly in treating neuronal hearing loss.

## Conflict of interest

There is no conflict of interest that could be perceived as prejudicing the impartiality of the research reported.
